# Occult cancer in patients with unprovoked venous thromboembolism: A nested case-control study

**DOI:** 10.1093/ajcp/aqad178

**Published:** 2024-02-10

**Authors:** Verónica Sánchez-López, Samira Marín-Romero, Marta Ferrer-Galván, Teresa Elías-Hernández, José Luis Lobo Beristain, Aitor Ballaz Quincoces, Luis Jara-Palomares, Francisco Javier Rodríguez Martorell, María José Castro, Carmen Marín Hinojosa, José Luis López-Campos, Remedios Otero-Candelera

**Affiliations:** Instituto de Biomedicina de Sevilla, Hospital Universitario Virgen del Rocio/CSIC/Universidad de Sevilla, Seville, Spain; Centro de Investigación Biomédica en Red de Enfermedades Respiratorias, Madrid, Spain; Medical Surgical Unit of Respiratory Diseases, Hospital Universitario Virgen del Rocío, Seville, Spain; Medical Surgical Unit of Respiratory Diseases, Hospital Universitario Virgen del Rocío, Seville, Spain; Instituto de Biomedicina de Sevilla, Hospital Universitario Virgen del Rocio/CSIC/Universidad de Sevilla, Seville, Spain; Centro de Investigación Biomédica en Red de Enfermedades Respiratorias, Madrid, Spain; Medical Surgical Unit of Respiratory Diseases, Hospital Universitario Virgen del Rocío, Seville, Spain; Hospital Universitario Araba, Vitoria-Gasteiz, Vizcaya, Spain; Hospital de Galdakao, Vizcaya, Spain; Instituto de Biomedicina de Sevilla, Hospital Universitario Virgen del Rocio/CSIC/Universidad de Sevilla, Seville, Spain; Centro de Investigación Biomédica en Red de Enfermedades Respiratorias, Madrid, Spain; Medical Surgical Unit of Respiratory Diseases, Hospital Universitario Virgen del Rocío, Seville, Spain; Hematology Department, Hospital Universitario Virgen del Rocío, Seville, Spain; Instituto de Biomedicina de Sevilla, Hospital Universitario Virgen del Rocio/CSIC/Universidad de Sevilla, Seville, Spain; Instituto de Biomedicina de Sevilla, Hospital Universitario Virgen del Rocio/CSIC/Universidad de Sevilla, Seville, Spain; Centro de Investigación Biomédica en Red de Enfermedades Respiratorias, Madrid, Spain; Instituto de Biomedicina de Sevilla, Hospital Universitario Virgen del Rocio/CSIC/Universidad de Sevilla, Seville, Spain; Centro de Investigación Biomédica en Red de Enfermedades Respiratorias, Madrid, Spain; Medical Surgical Unit of Respiratory Diseases, Hospital Universitario Virgen del Rocío, Seville, Spain; Instituto de Biomedicina de Sevilla, Hospital Universitario Virgen del Rocio/CSIC/Universidad de Sevilla, Seville, Spain; Centro de Investigación Biomédica en Red de Enfermedades Respiratorias, Madrid, Spain; Medical Surgical Unit of Respiratory Diseases, Hospital Universitario Virgen del Rocío, Seville, Spain

**Keywords:** occult cancer, D-dimer, P-selectin, extracellular vesicles, cell-derived microparticles, cancer biomarkers

## Abstract

**Objectives:**

Detecting occult cancer in patients with unprovoked venous thromboembolism (VTE) remains a significant challenge. Our objective was to investigate the potential predictive role of coagulation-related biomarkers in the diagnosis of occult malignancies.

**Methods:**

We conducted a nested case-control study with a 1-year prospective cohort of 214 patients with unprovoked VTE, with a focus on identifying occult cancer. At the time of VTE diagnosis, we measured various biomarkers, including soluble P-selectin (sP-selectin), dimerized plasmin fragment D (D-dimer), platelets, leukocytes, hemoglobin, total extracellular vesicles (EVs), EVs expressing tissue factor on their surface (TF+EVs), and EVs expressing P-selectin on their surface (Psel+EVs) in all participants.

**Results:**

We observed statistically significant increased levels of sP-selectin (*P* = .015) in patients with occult cancer. Despite an increase in Psel+EVs, TF+EVs, D-dimer, and platelets within this group, however, no significant differences were found. When sP-selectin exceeded 62 ng/mL and D-dimer surpassed 10,000 µg/L, the diagnosis of occult cancer demonstrated a specificity of up to 91% (95% CI, 79.9%-96.7%).

**Conclusions:**

The combination of sP-selectin and D-dimer can be a valuable biomarker in detecting occult cancer in patients with unprovoked VTE. Further research is necessary to ascertain whether easily measurable biomarkers such as sP-selectin and D-dimer can effectively distinguish between patients who have VTE with and without hidden malignancies.

**Key points**

- A bidirectional relationship exists between malignancies and thrombosis, with a notable percentage of patients with unprovoked venous thromboembolism (VTE) having a subsequent cancer diagnosis.
- Identifying patients with VTE who are at a higher risk of occult cancer poses a clinical challenge.
- Our study revealed that soluble P-selectin and dimerized plasmin fragment D constitute valuable biomarkers for detecting occult cancer in patients with unprovoked VTE.

## INTRODUCTION

Patients with a cancer diagnosis are at an increased risk of venous thromboembolism (VTE), which includes deep vein thrombosis (DVT) and pulmonary embolism (PE). Research suggests that approximately 2% to 15% of all patients with malignancies experience thrombotic events.^1-3^ It has been documented that in certain instances, VTE may present as the first sign of cancer. In fact, up to 10% of patients with an unprovoked VTE have subsequently diagnosed malignancies following their initial thrombotic event.^4,5^ To aid in the early detection of hidden cancers in patients with unprovoked VTE, clinical practice guidelines advocate for limited cancer screening that typically includes a review of the patient’s medical history, a physical examination, basic laboratory tests, and chest x-ray.^6^ Recent studies indicate, however, that implementing an extensive cancer screening, including more diagnostic tests, may have clinical significance for select patients. Additionally, some authors have developed a novel risk-prediction score to identify patients with unprovoked VTE who are at high risk for occult cancer and would benefit from more extensive screening.^7,8^ Although this score has yielded promising results, further research is necessary before it can be implemented in a clinical setting.

Numerous hypotheses have been put forward to explain the increased risk of thrombosis in patients with cancer. Cancer cells are known to express procoagulant molecules, such as tissue factor (TF), that possess the capacity to activate coagulation mechanisms directly.^9^ Furthermore, tumor cells can stimulate cytokine production, which in turn activates coagulation processes and fosters rapid cancer development.^10-13^ During the evolution of malignancies, the expression of P-selectin is enhanced. This protein promotes the adhesion of circulating malignant cells to leukocytes and activated platelets.^14^

Extracellular vesicles (EVs) are a diverse group of lipid bilayer–delimited particles that most cell types release naturally. Although they cannot replicate like cells do, they play a crucial role as mediators of cell-to-cell communication.^15^ Moreover, they have shown potential as biomarkers for the diagnosis and prognosis of various diseases, including thrombosis and cancer.^16-18^ The objective of this nested case-control study, conducted in a 1-year prospective cohort of patients experiencing unprovoked VTE, was to investigate the potential predictive value of biomarkers related to blood coagulation in identifying the presence of occult malignancies.

## METHODS

### Study Design

We conducted a nested case-control study in a prospective cohort of patients with unprovoked VTE recruited from 2 Spanish hospitals between 2012 and 2016. The study received approval from the Ethics Committee of our hospital, and all participants provided written informed consent. Our research strictly adhered to the principles outlined in the Declaration of Helsinki.

### Patients

#### Cohort Description: Patients With Unprovoked VTE

Consecutive patients who were diagnosed with their first confirmed episode of acute unprovoked VTE were monitored and followed-up for 1 year. Blood samples were collected on the same day as the VTE diagnosis. Patients who had begun anticoagulation therapy based on clinical suspicion of VTE more than 72 hours before the diagnosis were not included in the study. Individuals with a current or suspected cancer diagnosis; a history of neoplasia; or the presence of potential risk factors for thrombosis, such as recent surgery, prolonged immobilization by medical prescription, trauma, or hormone therapy, were also excluded.

#### Study Population: Selection of Case and Control Patients

Cases were selected from the unprovoked VTE cohort. Specifically, we selected those patients who received an objective cancer diagnosis during the follow-up period, occurring at least 30 days after unprovoked VTE diagnosis. As a comparative group, control patients were chosen from among those with unprovoked VTE who remained cancer free. To ensure balanced analysis, each case was meticulously matched with 3 controls, ensuring nonsignificant differences in terms of age and sex.

### Variables

We gathered demographic information and comorbidity data from all participants, including specifics about the type of VTE they had experienced—DVT or PE. Throughout the 12-month follow-up period, we collected data on key outcome measures such as all-cause mortality and cancer diagnoses. In addition, we conducted thrombophilia screening for patients with apparently unprovoked VTE younger than 50 years of age or those with a previous family or personal history of thrombosis. This standardized approach aligned with the routine clinical procedure followed at our hospital. Confirmation of acute symptomatic VTE was achieved through ultrasonography for suspected DVT and multidetector computed tomography scans for suspected PE. Histologic confirmation was required for cancer diagnosis. Additionally, blood samples from the study population were analyzed for dimerized plasmin fragment D (D-dimer); soluble P-selectin (sP-selectin); EV subpopulations; and various hemogram parameters, including leukocyte, platelet, and hemoglobin levels.

### Blood Sampling and Biomarker Testing

Venous blood samples were collected at baseline from all participants using a 21-gauge needle. The blood was drawn into 3.5-mL VACUETTE 9NC coagulation 3.2% trisodium citrate (0.109 mol/L) tubes (Greiner Bio-One). All tubes were kept in a vertical position with no agitation during transportation to the laboratory. The samples were processed within 2 hours of extraction. Platelet-poor plasma (PPP) was obtained by centrifugation, as our group described previously.^19^ In brief, the samples underwent centrifugation at 1500*g* for 30 minutes at 4 °C, with no brake application. The resulting PPP was then collected and stored at –80 °C for future use. The levels of D-dimer and sP-selectin in the PPP of both the case and control groups were measured using commercially available kits. The INNOVANCE D-Dimer Assay (Siemens Healthineers) was used to measure D-dimer, with a lower detection limit of 0.19 mg/L fibrinogen-equivalent units. For sP-selectin, the Human sP-selectin/CD62P Immunoassay (R&D Systems) was used according to the manufacturer’s guidelines. The results were expressed as µg/L for D-dimer and ng/mL for sP-selectin, respectively. The assessment of blood cell count, platelets, and hemoglobin levels was performed using standardized clinical laboratory procedures with a Sysmex XN-10 Automated Hematology Analyzer (Sysmex).

### EV Determination

Total EVs, EVs expressing TF on their surface (TF+EVs), and EVs expressing P-selectin on their surface (Psel+EVs) were quantified using a BD LSR II flow cytometer (BD Biosciences), as previously described by our group, with minor modifications.^20,21^ In brief, a total of 30 µL of PPP was thawed and incubated at a temperature range of 20 to 25 °C for 30 minutes in the dark. This incubation was carried out with the following specific monoclonal antibodies: mouse anti-human CD142-FITC (clone VD8, product No. 4508CJ; American Diagnostica) and mouse anti-human CD62-PE (clone AK-4, product No. 561921; BD Biosciences). To serve as negative controls, samples were also incubated with their isotype-matched negative control antibodies. Following the antibody incubation, 2 µL annexin V–CF Blue (Immunostep) and 20 µL annexin V binding buffer (10 mM HEPES, 140 mM NaCl, 2.5 mM CaCl_2_, pH 7.4) was added to each sample. The specimens were then incubated for 15 minutes at room temperature while being kept in the dark. Subsequently, 470 µL binding buffer was added to each sample, and the specimens were immediately subjected to flow cytometric analysis. To limit background noise from dust and crystals, all reagents were filtered twice using 0.22-µm filters. Additionally, to prevent fluorescent particle aggregation in the antibody solution, all antibodies, isotype controls, and annexin V were centrifuged at 16,000*g* for 10 minutes at 4 °C.^22^

**FIGURE 1:**
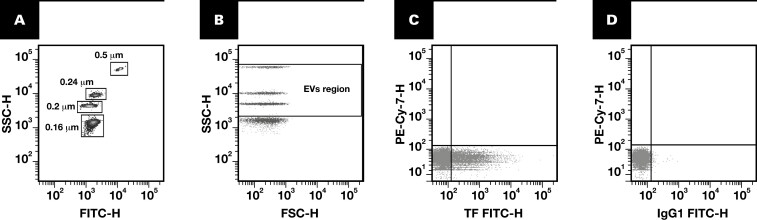
Identification of extracellular vesicles by flow cytometry. Calibration of a flow cytometer using Megamix-Plus SSC beads to set up gate limits for EV detection. **A**, Identification of bead subpopulations based on their complexity characteristics and fluorescent intensity. **B**, The establishment of EV gates at 0.5 µm (upper cloud), 0.24 µm (second cloud), 0.20 µm (third cloud), and 0.16µm (bottom cloud). The defined region for EVs (enclosed within 2 horizontal lines) encompasses the majority of the theoretical large EV size range (0.1-1 µm). **C**, **D**, Representative dot plots illustrating the EV subpopulation from a patient diagnosed with venous thromboembolism without cancer. **C**, Features of EVs carrying TF identified in the bottom right region with a TF marker (CD142^+^), with analogous isotype-matched negative control antibody (**D**). EV, extracellular vesicle; FITC, fluorescein isothiocyanate; H, height; IgG1, immunoglobulin G1; SSC, side scatter; TF, tissue factor.

Flow cytometer calibration was carried out using a mixture of fluorescent calibration beads (0.16, 0.20, 0.24, and 0.5 µm; Megamix-Plus SSC, Biocytex). These beads were specifically designed to establish an EV region in flow cytometers optimized for side scatter (SSC), such as the BD LSR II analyzer. The Megamix-Plus SSC protocol uses SSC as a size-related parameter and is designed to cover a significant portion of the theoretical range of large EV sizes (0.1-1 µm) Figure 1A, 1B.^20,23^ Total EVs were identified as flow cytometer events within the EV region and positive for annexin V, whereas TF+EVs and Psel+EVs were defined as events that were part of total EVs and immunoreactive to mouse anti-human CD142-FITC and mouse anti-human CD62-PE, respectively Figure 1C, 1D. For absolute quantification of EVs, counting spheres (6 µm) with a known concentration (approximately 1000 spheres/µL [Perfect-Count Microspheres, Cytognos]) were added to each sample as an internal standard. The samples were processed for 2 minutes at a low flow rate, with the results expressed as events per µL.

### Statistical Analysis

Categorical variables were presented as absolute values and percentages; continuous variables were represented using medians and interquartile ranges (IQRs). An exception was made for platelet count, however, which was also expressed as percentiles. To compare results between groups, we employed either the Student *t* test or the Mann-Whitney *U* test, as appropriate. Correlations, when evaluated, were conducted using the Spearman ρ. We derived receiver operating characteristic curves and expressed the likelihood of an occult cancer diagnosis following an unprovoked VTE in terms of sensitivity, specificity, positive predictive value, negative predictive value (NPV), and positive and negative likelihood ratios, based on biomarker cutoff points. We conducted all statistical analyses in SPSS, version 22.0, for Windows (IBM). Two-tailed *P *< .05 was considered statistically significant.

## RESULTS

### Study Population

**FIGURE 2:**
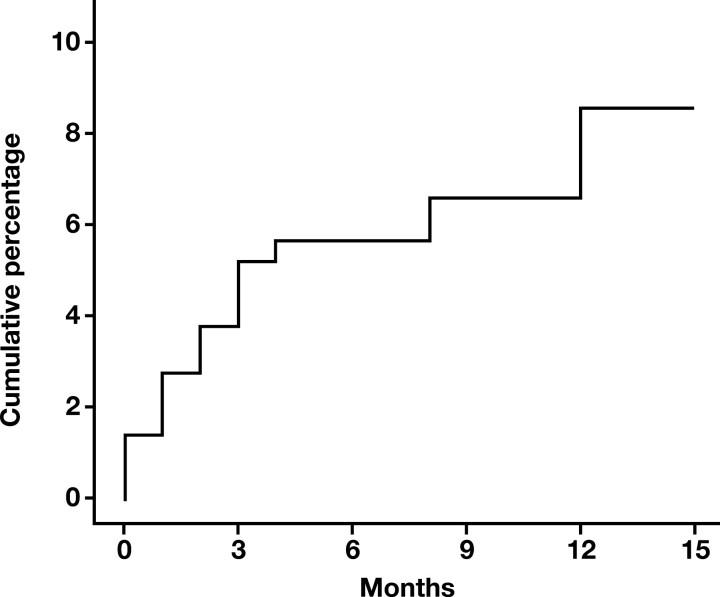
Cumulative incidence of cancer within the cohort of patients with unprovoked venous thromboembolism during the follow-up period. Results are depicted as percentages.

From 2012 to 2016, we enrolled 214 patients with unprovoked VTE and monitored them for 1 year. During the follow-up period, 19 patients were identified with cancer 30 days after diagnosis of unprovoked VTE, which suggests a cumulative cancer incidence of 8.8% (95% CI, 5.8%-13.4%) Figure 2 following unprovoked VTE events. Three patients were not included in the study because of a cancer diagnosis within the first month after VTE detection. Of the remaining 192 patients without diagnosis of malignancies, we selected 57 who were age and sex matched with case patients, to serve as the control group. Table 1 shows the clinical and demographic characteristics of the study groups. Table 2 outlines the details of occult cancer diagnosis, including the localization, stage, and histology of detected malignancies, as well as the time elapsed between VTE diagnosis and occult cancer detection. Analysis revealed that the majority of diagnoses were made during the first 3 months (11 new cases) and the last month (5 new cases) of follow-up. The initial VTE cohort primarily consisted of obese middle-aged and older (> 45 years of age) men with a VTE diagnosis. Additionally, these individuals typically presented with clinical signs of DVT in the lower limbs rather than PE. The final study population, which included cases and controls, showed similar characteristics to the initial cohort, except for being older and having a higher prevalence of arterial hypertension Table 1. In both groups, 65 participants, accounting for 85.5% of the total, underwent thrombophilia screening. Of these, 6 individuals (9.2%) had a prothrombotic genetic alteration. Among these alterations, 4 (6.1%) were factor V Leiden heterozygous variants, while 2 (3.1%) were factor II G20210A heterozygous variants. Additionally, 1 patient (1.5%) presented with S protein deficiency, 7 patients (10.8%) exhibited positive lupus anticoagulant or elevated cardiolipin levels, and 4 patients (6.1%) were identified with multiple hereditary thrombophilia.

**TABLE 1:** Patient Demographic and Clinical Characteristics

Characteristic	VTE cohort (n = 214)	Study population (n = 76)	Cases (n = 19)	Controls (n = 57)
Male sex, No. (%)	127 (59.3)	43 (56.6)	11 (57.9)	32 (56.1)
Age, median (IQR), y	67 (54-76)	75 (64-80)^a^	76 (64-79)	76 (63-80)
Body mass index, median (IQR), kg/m^2^	30.15 (27-33)	30.33 (26-33)	31.01 (28-34)	30.22 (28-33)
Smoker, No. (%)	72 (33.6)	29 (38.2)	8 (42.1)	21 (36.8)
Alcohol consumption, No. (%)	16 (7.5)	9 (11.8)	1 (5.3)	8 (14.0)
DVT, No. (%)	178 (83.2)	65 (85.5)	17 (89.5)	48 (84.2)
PE, No. (%)	13 (6.1)	4 (5.3)	1 (5.3)	3 (5.3)
DVT + PE, No. (%)	23 (10.7)	7 (9.2)	1 (5.3)	6 (10.5)
History of VTE, No. (%)	15 (7.0)	4 (5.3)	1 (5.3)	3 (5.3)
Arterial hypertension, No. (%)	108 (50.5)	48 (63.2)^b^	11 (57.9)	37 (64.9)
Dyslipidemia, No. (%)	56 (26.2)	26 (34.2)	4 (21.1)	22 (38.6)
Chronic lung disease, No. (%)	45 (21.0)	17 (22.4)	4 (21.1)	13 (22.8)
Diabetes, No. (%)	30 (14.0)	15 (19.7)	3 (15.8)	12 (21.0)
Heart disease,^c^ No. (%)	14 (6.54)	7 (9.2)	2 (10.5)	5 (8.8)
Chronic renal insufficiency, No. (%)	11 (5.1)	6 (7.9)	1 (5.3)	5 (8.8)
Mental disorders,^d^ No. (%)	30 (14.0)	13 (17.1)	2 (10.5)	11 (19.3)
Other comorbidities,^e^ No. (%)	26 (12.1)	7 (9.2)	3 (15.8)	4 (7.0)

DVT, deep vein thrombosis; IQR, interquartile range; PE, pulmonary embolism; VTE, venous thromboembolism.

^a^ *P* < .001.

^b^ *P* < .005.

^c^Encompasses conditions such as heart failure and ischemic heart disease.

^d^Encompasses depression, anxiety, and psychotic and bipolar disorders.

^e^Includes thyroid disorders, hepatopathy, dyspeptic syndrome, and connective tissue diseases.

**TABLE 2:** Characteristics of Occult Malignancies Identified in Study Patients

	Cancer			
Patient	Localization	Stage	Histology	Interval between VTE diagnosis and detection of occult cancer, d
1	Biliary tract	IV	Adenocarcinoma	341
2	Pleura	IV	Adenocarcinoma	73
3	Acute myeloblastic leukemia	—	—	93
4	Hepatocellular carcinoma	IA	Clear cell component	264
5	Colon	IIA	Adenocarcinoma	151
6	Lung	IV	Adenocarcinoma	261
7	Prostate	IV	Adenocarcinoma	34
8	Bladder	IIIB	Squamous differentiation	31
9	Colon	IV	Adenocarcinoma	48
10	Prostate	IIA	Adenocarcinoma	85
11	Uterus	IIIB	Adenocarcinoma	38
12	Colon	IIA	Adenocarcinoma	106
13	Lung	IIIB	Adenocarcinoma	43
14	Uterus	IIA	Adenocarcinoma	79
15	Colon	IIA	Adenocarcinoma	429
16	Lung	IIB	Adenocarcinoma	114
17	Prostate	IIA	Adenocarcinoma	38
18	Lung	IIA	Adenocarcinoma	112
19	Multiple myeloma	IIIA		142

VTE, venous thromboembolism.

### Blood Biomarkers

We measured sP-selectin; D-dimer; and platelet, leukocyte, and hemoglobin levels in both cases and controls. In patients with VTE who subsequently developed cancer, we noticed a nearly 2-fold increase in sP-selectin level compared with those who did not develop malignancies (median [IQR], 72 [39-86] ng/mL vs 41 [28-59] ng/mL; *P* = .015) Table 3. There was a slightly elevation in median D-dimer levels in patients who developed cancer, but this difference failed to achieve statistical significance (median [IQR], 4596 [3254-18,853] µg/L vs 3882 [2612-8472] µg/L; *P* = .113) Table 3. In relation to leukocyte, platelet, and hemoglobin levels, no statistically significant differences were observed when the absolute values were compared across different groups Table 3. When patients in our study population were divided into groups based on diagnosis of leukocytosis (cutoff, 11 × 10^9^ leukocytes/L) or platelet counts equal to or above the 90th percentile (350 × 10^3^ platelets/µL), interesting observations were made. Specifically, among patients with VTE and platelet counts equal to or above the 90th percentile, 62.5% (5/8) were later diagnosed with cancer. This finding indicates that patients with platelet counts equal to or above 350 × 10^3^ platelets/µL accounted for more than 25% of patients with VTE who developed cancer, whereas only 5.3% of thrombotic patients without malignancies exceeded this cutoff (*P* = .003). No significant difference was found between groups based on the presence or absence of leukocytosis.

**TABLE 3:** Biomarker Data

	Study population (n = 76)	Cases (n = 19)	Controls (n = 57)	*P* value^a^
Total EVs, events/µL, median (IQR)	7110 (5105-10,674)	7820 (5520-13,278)	6992 (5520-13,278)	.280
Psel+EVs, events/µL, median (IQR)	727 (401-1034)	852 (618-1018)	696 (370-1034)	.295
TF+EVs, events/µL, median (IQR)	32 (18-56)	29 (22-51)	36 (16-56)	.773
Hemoglobin, g/L,median (IQR)	134 (114-147)	124 (110-142)	135 (115-149)	.221
Leukocytes, ×10^6^/L, median (IQR)	797 (658-925)	820 (678-951)	796 (625-918)	.674
Leukocytes >11 × 10^9^/L, No. (%)	8 (10.5)	3 (15.8)	5 (8.8)	.666
Platelets, ×10^3^/µL, median (IQR)	238 (193-289)	261 (205-324)	233 (190-288)	.331
Platelets ≥350 × 10^3^/µL, No. (%)	8 (10.5)	5 (26.3)	3 (5.3)	.003
D-dimer, µg/L, median (IQR)	4204 (2729-10,300)	4596 (3254-18,853)	3882 (2612-8472)	.113
sP-selectin, ng/mL, median (IQR)	46 (32-62)	72 (39-86)	41 (28-59)	.015

D-dimer, dimerized plasmin fragment D; EV, extracellular vesicle; Psel+EV, extracellular vesicle expressing P-selectin; IQR, interquartile range; sP-selectin, soluble P-selectin; TF+EV, extracellular vesicle expressing tissue factor.

^a^ *P* values for comparisons of cases vs controls.

### EV Quantification

We measured total EVs, TF+EVs, and Psel+EVs in cases and controls. No significant differences were found between the 2 groups Table 3. When analyzing EVs expressing P-selectin on their surface, however, patients with both VTE and cancer showed higher median values than those with VTE but no cancer (median [IQR], 852 [618-1018] events/µL vs 696 [370-1034] events/µL; *P* = .295). Conversely, EVs expressing TF showed higher median values in patients with VTE but no cancer than those with both VTE and cancer (median [IQR], 36 [16-56] events/µL vs 29 [22-51] events/µL; *P* = .773).

### Distribution of Blood Coagulation Biomarkers and EVs in Cases and Controls

**FIGURE 3:**
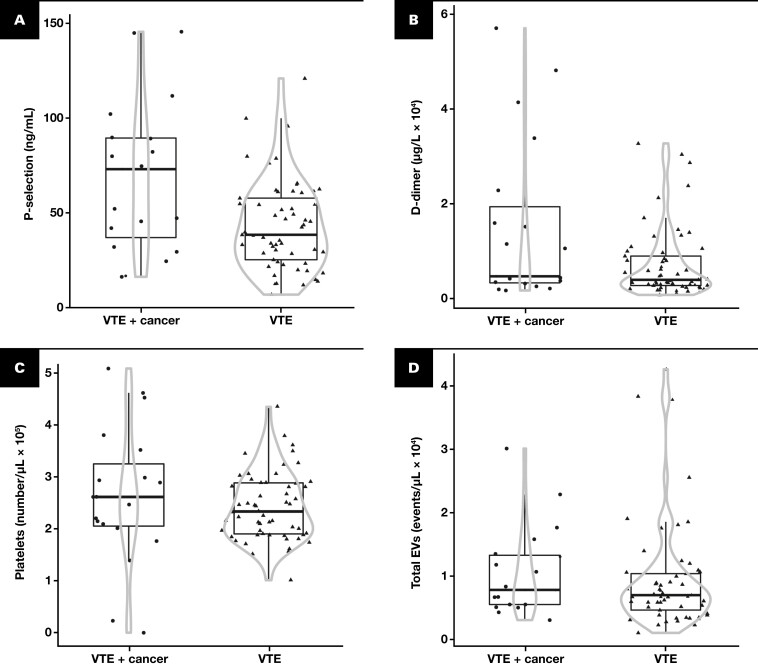
Violin chart showing a comparison of biomarkers in patients diagnosed with VTE and VTE with occult cancer. The biomarkers included soluble P-selectin (**A**), D-dimer (**B**), platelets (**C**), and total EVs (**D**). Results are expressed as medians (indicated by the horizontal line inside the box) and interquartile ranges (IQRs) (upper and lower horizontal lines defining the box) and 1.5-fold IQRs (whiskers). The circle represents patients with VTE and occult cancer, while the triangle denotes patients with VTE but not cancer. The gray lines demarcate the density area, with the width directly proportional to the frequency. D-dimer, dimerized plasmin fragment D; EV, extracellular vesicle; VTE, venous thromboembolism.

We selected specific biomarkers—namely, D-dimer, sP-selectin, platelet count, and total EVs—based on our findings of differences between groups. Our results revealed that patients with a cancer diagnosis exhibited a greater dispersion of data in all cases than patients with VTE but no cancer diagnosis Figure 3. These observations suggest that patients with VTE and occult malignancies display a greater spread of data than those without cancer, irrespective of the biomarker analyzed.

### D-dimer, sP-Selectin, and Platelets as Potential Predictors of Occult Cancer in Patients With VTE

Based on the variations and data distribution observed in prior analyses, we selected sP-selectin, D-dimer, and platelets as potential biomarkers to accurately detect or dismiss cancer diagnoses. We computed receiver operating characteristic curves and selected the optimal cutoff points that delivered the highest sensitivity and specificity. Patients were then categorized into subgroups based on the following cutoff points: greater than 30 ng/mL or greater than 62 ng/mL for sP-selectin, exceeding 10,000 µg/L for D-dimer and 350 × 10^3^/µL or more for platelets. We specifically chose 2 cutoff points for sP-selectin upon identifying values that demonstrated high sensitivity and high specificity. Our aim was to investigate the predictive potential of these 2 points, particularly when combined with other parameters. The results revealed that patients with VTE and occult cancer had the highest levels of sP-selectin and D-dimer. Interestingly, more than 50% of patients with cancer had sP-selectin levels exceeding 62 ng/mL, whereas only 15.8% of patients without malignancies exhibited similar concentrations (*P* = .003). Moreover, 47% of patients with VTE diagnosed with cancer had D-dimer levels above 10,000 µg/L compared with 21% in the noncancer VTE group (with a difference of borderline significance at *P* = .054).

In terms of sensitivity and specificity, we found that sP-selectin at its lowest cutoff values exhibited a high sensitivity of 82.2%. This sensitivity decreased, however, when the highest cutoff point was used or when sP-selectin was combined with the D-dimer cutoff point Table 4. Conversely, the specificity achieved at the highest sP-selectin values was remarkable: A cutoff point exceeding 62 ng/mL demonstrated a specificity of 84.2%. This specificity increased to 91% when a D-dimer cutoff above 10,000 µg/L was included Table 4. Additionally, the analysis of predictive value revealed a high NPV. The cut-off for P-selectin greater than 62 ng/mL yielded an NPV of 84.2%, which remained consistent when a D-dimer cutoff exceeding 10,000 µg/L was implemented, resulting in an NPV of 82.5% Table 4.

**TABLE 4:** Sensitivity, Specificity, and Predictive Values of Soluble P-Selectin and D-Dimer in Detecting Occult Cancer

	Soluble P-selectin >30 ng/mL, % (95% CI)	Soluble P-selectin >62 ng/mL, % (95% CI)	D-dimer >10,000 µg/L, % (95% CI)	Soluble P-selectin >30 ng/mL and D-dimer >10,000 µg/L, % (95% CI)	Soluble P-selectin >62 ng/mL and D-dimer >10,000 µg/L, % (95% CI)	Platelet count ≥350 × 10^3^/µL, % (95% CI)
Sensitivity	82.2 (62.4-94.4)	52.6 (29.5-74.7)	47.3 (27.3-68.3)	47.3 (25.2-70.5)	42.1 (21.1-66.1)	26.3 (11.8-48.8)
Specificity	26.3 (16.5-38.9)	84.2 (71.6-92.1)	78.9 (66.7-87.5)	82.4 (69.6-90.8)	91.2 (79.9-96.7)	94.7 (85.6-98.2)
PPV	27.6 (17.0-41.1)	52.6 (29.5-74.7)	42.8 (22.6-65.6)	47.3 (25.2-70.5)	61.5 (32.3-84.9)	62.5 (30.6-86.3)
NPV	83.3 (57.7-95.5)	84.2 (71.6-92.1)	81.8 (68.6-90.5)	82.4 (69.6-90.8)	82.5 (70.5-90.6)	79.4 (68.4-87.3)
Patients correctly diagnosed	40.8 (29.8-52.7)	76.3 (64.9-85.0)	71.0 (62.1-82.8)	73.7 (62.1-82.8)	78.9 (67.8-87.1)	77.6 (67.1-85.5)
Diagnostic odds ratio	1.90 (0.48-7.47)	5.92 (1.88-18.7)	3.37 (1.12-10.16)	4.23 (1.4-13.1)	7.56 (2.0-27.5)	6.6 (2.8-14.9)

NPV, negative predictive value; PPV, positive predictive value.

Furthermore, there was a notable correlation between the odds ratio (OR) and elevated levels of sP-selectin, particularly in conjunction with the D-dimer cutoff—notably, when sP-selectin levels surpassed 30 ng/mL the OR rose to 1.90 (95% CI, 0.48-7.47)—and this increase was even more pronounced when accompanied by a D-dimer level exceeding 10,000 µg/L with the OR reaching 4.23. Moreover, sP-selectin values higher than 62 ng/mL demonstrated an OR of 5.92, which increased to 7.56 when associated with a D-dimer concentration above 10,000 µg/L. Regarding platelet count, the 350 × 10^3^ platelets/µL cutoff point proved to be effective in differentiating between patients with VTE and hidden malignancies and those without cancer. It demonstrated an excellent specificity of 94.7% (95% CI, 85.6%-98.2%), accompanied by a commendable NPV of 79.4% (95% CI, 68.4%-87.3%) and an OR of 6.6 (95% CI, 2.8%-14.9%) Table 4.

## DISCUSSION

The current investigation assessed the potential utility of parameters linked to blood coagulation and procoagulant EVs as predictive biomarkers for occult cancer within a group of patients experiencing unprovoked VTE. Our findings indicated that biomarkers such as sP-selectin in combination with D-dimer could aid in identifying patients with VTE and hidden neoplasms.

In our study, we documented a cumulative incidence of cancer following unprovoked VTE events of 8.8% (95% CI, 5.8%-13.4%) during a 1-year follow-up period. It is important to note that the reported incidence of VTE-associated cancer can vary significantly among studies, primarily because of variations in ascertainment methods and the characteristics of the populations studied. For instance, a review conducted in 2008 examined 36 studies focusing on occult cancer in patients with newly diagnosed VTE. The authors found that the prevalence of hidden cancer was 6.1% at baseline and increased to 10.0% at 12 months after the diagnosis of VTE.^4^ A meta-analysis published in 2017 that included 10 studies and analyzed data from 2316 patients revealed that approximately 5.2% of patients were diagnosed with occult cancer within the first year after a VTE episode.^24^ Some authors indicated that the annual incidence of VTE in patients with different types of cancer is approximately 10.9%. This percentage rises to 15% when considering patients with colorectal cancer treated with a combination of fluorouracil and leucovorin calcium.^1^ Notwithstanding the existing discrepancies, the majority of studies documented a cancer incidence rate among patients with VTE ranging from 3% to 10%, a finding that aligns with the 8.8% incidence observed in our investigation.

D-dimer and sP-selectin play a crucial role in predicting thrombosis in patients with malignancies. These biomarkers have shown significant improvements in enhancing the accuracy of the Khorana risk score, a predictive model specifically designed to identify the risk of VTE in individuals diagnosed with cancer.^25^ The role of D-dimer or sP-selectin, however, as markers of occult cancer in patients with VTE is not well understood. Our study revealed that sP-selectin alone or in combination with D-dimer can effectively differentiate between patients with unprovoked VTE who developed cancer and those who did not with a remarkable specificity (91%) and a high NPV (82%). Although investigations into sP-selectin have been limited, the existing studies support our findings. For instance, a recent cohort study involving 22 patients with VTE and cancer and 347 patients with VTE free from malignancies concluded that higher sP-selectin levels, age over 50 years, and lower plasma clot permeability were independent predictors of hidden cancer according to a multivariable Cox analysis.^26^ Other authors have proposed that prothrombotic markers, including sP-selectin, could play a significant role in detecting cancer in patients experiencing arterial thrombosis.^27^ Recent findings have also suggested a potential role for D-dimer in identifying VTE-associated cancer. A 2016 study found that the proportion of occult cancer in patients with unprovoked VTE was higher in patients with baseline D-dimer levels above 4000 ng/mL (32.0%) than in those with levels below 2000 ng/mL (9.3%) and 2000 to 4000 ng/mL (13.8%). The authors concluded that D-dimer levels above 4000 ng/mL were independently associated with occult cancer compared with levels below 2000 ng/mL.^28^ In a prospective investigation, a significant relationship was observed between D-dimer levels above 4000 ng/mL and cancer diagnosis in patients with DVT who were followed-up for an average of 36 months.^29^ Our study demonstrated the potential of D-dimer levels in identifying hidden cancer among patients with VTE. That we did not find a statistically significant difference in D-dimer absolute values between VTE patients with and without cancer was unexpected, although this finding aligns with recent reports describing this observation. In prospective cohort studies involving patients with unprovoked VTE, no significant differences in median values for D-dimer were observed when comparing patients who developed cancer with those who did not.^26,30^ The discrepancy can be partly explained by the limited sample size and the chosen strategy for statistical analysis. In our investigation, we opted to express D-dimer values as median (SE) because we believe that it is the most suitable centralization parameter for studies with this sample size.

Currently, 2 risk-prediction scales are available for identifying occult malignancies in patients with thrombosis: the Computerized Registry of Patients with Venous Thromboembolism (RIETE) and the Screening for Occult Malignancy in Patients with Idiopathic Venous Thromboembolism (SOME) scales.^8,31^ The predictive factors of occult neoplasia in both scales are based on simple characteristics, but the efficacy of these instruments in clinical practice has recently come under scrutiny.

In 2018, a group of researchers conducted the Hokusai-venous thromboembolic study to evaluate the performance of the 2 scores. The study followed 8032 patients for 1 year. The results indicated that the C statistics for the RIETE and SOME scales were 0.62 (95% CI, 0.57-0.66) and 0.59 (95% CI, 0.55-0.62), respectively. The cumulative incidence of cancer diagnosis during the follow-up period for patients classified as “high risk” for hidden malignancies was 2.9% (95% CI, 2.1%-3.9%) for the RIETE score and 2.7% (95% CI, 1.9%-3.7%) for the SOME score, corresponding to hazard ratios of 1.8 (95% CI, 1.3-2.5) and 1.5 (95% CI, 1.04-2.2), respectively.^32^ The underwhelming performance of the RIETE and SOME scales highlights the need for additional studies to enhance these predictive models. Our findings suggest that incorporating sP-selectin (62 ng/mL) and D-dimer (10,000 µg/L) as cutoff points would significantly enhance the effectiveness of these risk prediction scales.

Our study included variables such as age, sex, smoking status, presence of chronic lung disease, hemoglobin levels, and platelet count, which are also considered in the RIETE and SOME scales. Both risk scores acknowledge advanced age as a risk factor for occult cancer, which aligns with the findings of the present report (median [IQR], 67 [54-76] vs 75 [64-80]; *P* < .001). Furthermore, our observations indicate that male sex is not a risk factor for hidden neoplasia. It is worth noting that although the SOME scale does not consider sex, the RIETE score recognizes male sex as a risk factor for occult malignancies. Sex is a contentious factor that exhibits variations based on patient series and cancer histology. In a case-control study derived from a cohort of 5864 patients with VTE included in the RIETE registry, 444 individuals were subsequently diagnosed with cancer, 55% of whom were men. Notably, men exhibited a heightened susceptibility to lung cancer, whereas their risk of pancreatic cancer was comparatively lower than that of women.^33^ We hypothesize that men experiencing unprovoked VTE may possess an inherent susceptibility to specific subtypes of occult cancer. To substantiate this theory, however, it is crucial to conduct additional comparative studies between men with unprovoked VTE diagnosed with cancer and those with cancer but no history of VTE.

Conversely, our study assessed the values for hemoglobin, leukocytes, and platelets, observing no significant differences between cases and controls. We did, however, note a markedly elevated percentage of patients with VTE who developed cancer exhibiting platelet counts of 350 × 10^3^/µL or higher. This observation should be interpreted with caution, however, because of the limited sample size (5 patients with VTE and occult cancer and 3 patients with VTE but no cancer). In addition, it is important to note that anemia and thrombocytosis are considered predictive indicators for hidden malignancies according to the RIETE scale.^31^ The Khorana risk score incorporates both anemia and thrombocytosis as predictive factors for VTE in patients with cancer.^34,35^ Conversely, the SOME scale does not encompass any of these variables in its risk assessment framework.^8^

Finally, our study employed flow cytometry to quantify the levels of total EVs, Psel+EVs, and TF+EVs. Extracellular vesicles are gaining recognition as potential biomarkers for numerous diseases, including thrombosis and cancer, but the identification and characterization of EVs present substantial challenges because of the absence of standardized methods for preanalytical and analytical processes. Numerous techniques are used to determine EVs, including atomic force microscopy,^36^ nanotracking analysis,^37^ dynamic light scattering,^38^ and flow cytometry.^39,40^ A commonly preferred method for EV quantification is flow cytometry^21,41-43^ because of its ability to detect, quantify, and characterize EVs simultaneously. Furthermore, flow cytometry is widely available and relatively cost-effective. Nevertheless, analysis of EVs using flow cytometry presents considerable challenges,^41^ including low sensitivity to resolve the total number of EVs, high background noise, and problematic swarm detection, making absolute EV quantification difficult. Despite the technical limitations of flow cytometry, however, reliable and reproducible results of EV determination have been obtained when the measurements were obtained using different flow cytometers calibrated with size-calibrated polystyrene beads.^20,23,42^ Among the various aspects of EV characterization, the quantification of TF+EVs is particularly contentious, which are primarily evaluated using either immunologic (based on TF-antigen detection) or functional (based on TF-activity quantification) methods. Discrepancies often arise between these 2 techniques, however. One potential explanation for these inconsistencies could be the antibody’s interaction with “encrypted” or degraded forms of inactive TF protein, which results in a weak correlation between levels of TF protein and actual TF activity. Additionally, the presence of fluorescent particles in commercial antibody solutions is under consideration as a factor that could cause false-positive TF+EVs events in flow cytometry quantification.^22^ The current sensitivity level of antigen-based assays is believed to be inadequate to detecting low levels of TF in plasma accurately, which has led to a recommendation for functional TF analysis.^43^ Some researchers considered deficiency in the stringent criteria for the monospecificity of anti-TF antibodies,^44^ while other studies have shown that reliable detection of TF using commercial antibodies is possible, with the effectiveness depending on the specific antibody clone used. The TF9-10H10 and VD8 clones have been found to be effective for detecting TF on EVs through flow cytometry. Conversely, the CLB/TF-5 and VIC7 clones may exhibit nonspecific binding.^45^

In an effort to address flow cytometry limitations and controversies between authors, 3 international societies—the International Society for Extracellular Vesicles, the International Society for Advancement of Cytometry, and the International Society on Thrombosis and Haemostasis—have collaborated to form the EV Flow Cytometry working group.^46^ This task force is dedicated to overcoming these hurdles, with a primary goal to enhance the identification of EVs using flow cytometry.

In this study, we analyzed the levels of total EVs, Psel+EVs, and TF+EVs using flow cytometry. We were unable to establish Psel+EVs or TF+EVs as reliable predictors of occult cancer in patients with VTE under the applied technique and methodology. It is worth noting, however, that Psel+EV levels were slightly increased in patients with VTE and hidden malignancies, whereas TF+EV levels were marginally increased in thrombotic patients who did not develop cancer. These observations suggest that TF may be a potential biomarker for thrombosis, whereas sP-selectin expression could be more closely associated with underlying cancer. Our previous studies have provided further evidence supporting this notion. In a prospective investigation, we examined different phenotypes of EVs in a cohort made up of 138 patients with unprovoked VTE who did not develop cancer as well as 67 patients with advanced cancer who did not experience thrombosis during a 1-year follow-up period. Our findings revealed a significant increase in TF+EV levels in patients with VTE but no cancer compared with patients with cancer but not thrombosis. Furthermore, we observed that TF+EV levels were higher in patients with both VTE and cancer than in healthy individuals, although this difference reached statistical significance only in the case of patients with unprovoked VTE. These results strongly suggest that elevated levels of TF+EVs are characteristic of patients with VTE but not cancer.^21^

Therefore, we conclude that sP-selectin could be a valuable biomarker for detecting occult cancer in patients with unprovoked VTE. Incorporating sP-selectin, either alone or in combination with other biomarkers (eg, D-dimer), into predictive scores for hidden cancer risk would enhance their predictive accuracy. Further investigation is required to evaluate the role of platelet count and EVs as differentiating factors among thrombosis, cancer, and patients predisposed to developing these conditions.
